# Fulminant Necrotising Amoebic Colitis of Whole of Large Bowel: A Rare Complication of a Common Infectious Disease

**DOI:** 10.1155/2020/8845263

**Published:** 2020-08-11

**Authors:** Mohd Yasir Beg, Lovenish Bains, Ratnesh Mahajan, Pawan Lal, Sharmana Choudhury, N. Pritesh Kumar, Eva Wilse C. Momin, Veer Pal

**Affiliations:** ^1^Department of Surgery, Maulana Azad Medical College, New Delhi, India; ^2^Department of Pathology, Maulana Azad Medical College, New Delhi, India

## Abstract

**Background:**

Fulminant necrotising amoebic colitis (FulNAC) is an uncommon and grave complication of a very common infectious disease widely prevalent in tropical countries. In most of the cases reported, only a segment of large bowel was gangrenous. The involvement of the whole of the large bowel, as in our case, is very rare and has very high mortality ranging from 55% to 100%. *Case Summary*. A 50-year-old gentleman presented with an acute abdomen with a history of crampy abdominal pain and passage of blood mixed with mucous and loose stools. After resuscitation and investigations, the patient was taken up for laparotomy and the findings showed that the caecum was sloughed off and the entire large bowel had multiple perforations. Subtotal colectomy with ileostomy was performed. Histopathological examination showed evidence of pancolitis with multiple colonies of amoebic trophozoites. *Discussion*. *Entamoeba histolytica* is a protozoon that affects the large intestine and liver in humans. There can be various presentations of amoebiasis: asymptomatic infection (90%), symptomatic noninvasive infection (6–8%), acute amoebic colitis (dysentery), or fulminant colitis with perforation. FulNAC is an uncommon complication, difficult to diagnose and treat, and associated with a high mortality rate, ranging from 55% to 100%.

**Conclusion:**

It is important to consider the possibility of fulminant necrotising amoebic colitis (FulNAC) as an uncommon and fatal complication of amoebiasis, especially in tropical countries, where amoebiasis is prevalent. Early diagnosis and antiamoebic treatment, along with urgent aggressive surgical resection of the involved segment and exteriorization of the proximal and distal bowel ends, are shown to reduce mortality.

## 1. Introduction

Amoebiasis is a parasitic infection common in underdeveloped countries with poor sanitation, in many tropical and subtropical areas of the world. The estimated worldwide annual mortality associated with amoebiasis is around 100,000 [1]. There can be various presentations of amoebiasis: asymptomatic infection (90%), symptomatic noninvasive infection (6–8%), and acute amoebic colitis (dysentery). It principally affects the colon and liver, with the caecum and ascending colon being involved in 70% cases [2].

Very rarely (<0.5% cases) [3], the disease takes a fulminant course leading to the development of fulminant necrotising amoebic colitis, which causes gangrenous necrosis of the involved large bowel. In most of the cases reported, only a segment of the large bowel was involved; the involvement of the whole of the large bowel, as in our case is very rare. Despite advancements in the field of surgery, the outcome of FulNAC has not changed much over the decades. The mortality recorded in all cases reported until the 1970s was 100%. Even today, the mortality is very high ranging from 55% to 100% [4]. The main cause of death in such patients is septicemia arising as a complication of perforation peritonitis. We present an uncommon case of FulNAC involving the whole of the large bowel, where the patient was saved by early surgical intervention.

## 2. Case Presentation

A 50-year-old gentleman, resident of a slum area in the outskirts of New Delhi, presented to surgery emergency with complaints of diffuse abdominal pain for 5 days, obstipation for 2 days, and abdominal distension for 1 day. There was a history of recurrent episodes of loose stools mixed with mucous and blood associated with intermittent crampy lower abdominal pain for the past 7 days. The patient was a chronic alcoholic and a known case of diabetes mellitus. On examination, the patient was dehydrated, pulse rate was 120/min, and blood pressure was 90/60 mmHg, and was maintaining a saturation of 98% on room air. The abdomen was grossly distended and tenderness was present all over. Rectal examination done later revealed distended rectum and examining finger was tinged with blood and foul-smelling mucous.

**Figure 1:**
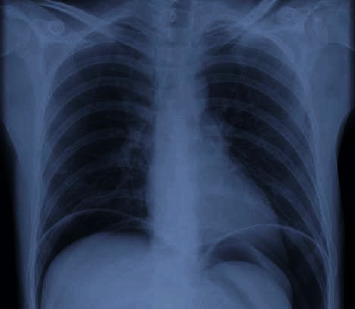
Chest X-ray showing gas under diaphragm.

The patient was resuscitated with intravenous fluids following which he improved to BP 100/72 mm Hg, PR 102/min, RR 17/min, and urine output of 200 ml over 4 hours. After stabilization, the patient was shifted for chest and abdominal X-ray, which showed gas under the diaphragm (Figure 1). ABG analysis showed mild metabolic acidosis which got corrected with fluid resuscitation. Hemoglobin was 8.2 gm%, total leucocyte counts were 24000/mm^3^, blood urea was 72 mg/dl, and serum creatinine was 1.8 mg/dl. Serum electrolytes and coagulation profile were within the normal range. (Na 136 mEq/L, K 4.2 mEq/L, INR 1.2).

**Figure 2:**
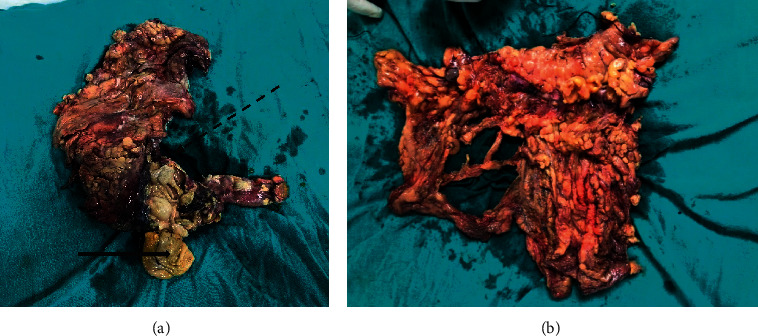
(a) Resected bowel segment showing the gangrenous ascending colon (dashed arrow) and sloughed off caecum (bold arrow). (b) Resected transverse colon with mesocolon (gangrenous with multiple perforations).

The patient was started on ceftriaxone and metronidazole as per our institutional protocol and amikacin was added after one day when KFTs became normal. The patient was taken up for emergency laparotomy. Approximately 1000 ml of feculent pyoperitoneum was present. The caecum was found to be gangrenous and had sloughed off (Figure 2) and there were multiple perforations in the ascending colon and transverse colon. Initially, right hemicolectomy was done but the resected end of the transverse colon showed multiple sloughed off ulcers in the mucosa with transmural involvement. The proximal sigmoid colon was also found to be perforated into the retroperitoneum. There was no evidence of thrombosis of mesenteric vessels. In view of intraoperative findings, the procedure was converted into subtotal colectomy with proximal ileostomy and exteriorization of the distal end of the sigmoid colon.

**Figure 3:**
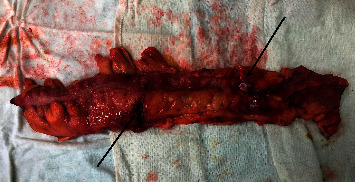
Resected descending colon showing multiple perforations (arrows).

**Figure 4:**
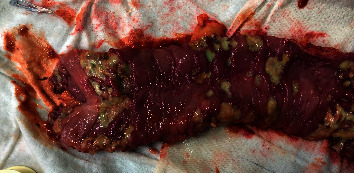
Opened up descending colon showing multiple areas of sloughed off mucosa with necrosis and perforation.

**Figure 5:**
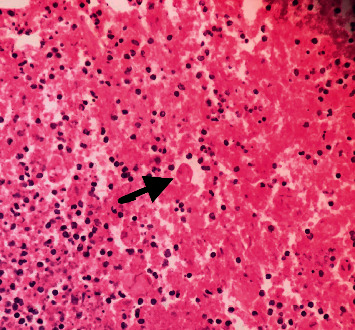
H&E stained tissue from ulcer from the resected specimen of the colon; arrow indicates trophozoites of *E. histolytica*.

**Figure 6:**
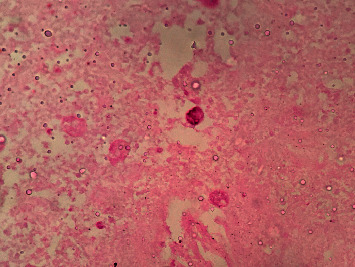
Periodic acid-Schiff (PAS) positive amoebic trophozoites.

Gross examination of the resected colectomy specimen showed sloughed off caecum with multiple perforations in the ascending, transverse, and descending colon (Figure 3). On cut section examination, there were patchy segments of extensive transmural necrosis with multiple defects ranging from 0.5–2 cm throughout the large bowel (Figure 4). Histopathological examination of the specimen showed findings of multiple mucosal ulcerations with liquefactive necrosis with normal intervening mucosa, associated with full-thickness perforations and extensive diffuse transmural inflammation. The ulcers were typically flask-shaped, with a narrow neck and broad, undermined base. In the colon, there was no evidence of architectural distortion or any evidence of crypt destructive colitis or granulomas to support the presence of Crohn's disease. The colonic mucosal ulcers were covered with neutrophilic debris within which *E. histolytica* were identified (Figure 5). Pathogenic Entamoebae trophozoites were recognized by the presence of phagocytosed ingested intracellular red cells. Periodic acid-Schiff staining was positive (Figure 6). Amoebic serology was positive with high IgM antibody titers (2.24) <0.04.

The patient had an uneventful recovery and was discharged on day 8 and was continued on oral metronidazole for 2 weeks and the luminal amoebicidal agent, diloxanide furoate, for 10 days. The patient underwent surgery for restoration of continuity of the bowel after 3 months and ileorectal anastomosis was done. The patient was healthy and symptom-free up to 2 months of follow-up in OPD.

## 3. Discussion

*Entamoeba histolytica* is a protozoon that affects the large intestine and liver in humans. Amoebiasis is seen predominantly in developing countries due to poor sanitation and increased faecal contamination of water supplies. Infection occurs due to ingestion of food or water contaminated by faeces [5]. In our case, the patient belonged to a slum area and usually these slums do not have proper access to the sanitation system, and the source of drinking water is also not safe. The infection may involve any part of the large bowel but there is a slight predilection for the caecum and ascending colon [6]. Around 50 million people contract the infection worldwide, with an estimated 40,000–100,000 deaths reported annually, making amoebiasis the second leading cause of death from parasitic diseases [7]. Prevalence is disproportionately higher and maybe as high as 50% in certain developing countries [8]. Amoebiasis affects about 15% of the Indian population and the incidence varies from 2 to 55% [9].

There can be various presentations of amoebiasis: asymptomatic infection (90%), symptomatic noninvasive infection (6–8%), acute amoebic colitis (dysentery), appendicitis, and ameboma or fulminant colitis with perforation [10]. The most fatal complication of intestinal amoebiasis is acute fulminant necrotising amoebic colitis. Such cases of virulent host response to amoebic infection in the form of FulNAC are rare (<0.5%) and only a few such cases have been reported in the literature [11]. According to a report from South Africa, acute fulminating colitis developed in only 3% of 3013 patients with amoebic colitis [1]. Colonic perforation is common in acute fulminant amoebic colitis, having an incidence of 30.4%. Single perforation is seen in majority cases, while multiple are also seen in one-third cases, usually involving the caecum and ascending colon [12]. In most of the cases reported, only a segment of the large bowel was involved; the involvement of the whole of the large bowel, as in our case is very rare.

Development of FulNAC is associated with various risk factors including male gender, extremes of age (>60 years and <10 years), progressive abdominal pain, associated liver abscess, malnutrition, immunocompromised state, immunosuppressants, leucocytosis, dyselectrolytemia, and hypoalbuminemia [13]. Peritonitis may develop either because of frank perforation due to transmural inflammation or necrosis leading to leaking through an extensively necrosed bowel [14]. Immunosuppression favours invasion of amoeba through the gut wall forming typical flask-shaped ulcers [15]. FulNAC is an uncommon complication, arduous to diagnose and treatment, and associated with a high mortality rate, ranging from 55% to 100% [16].

Diagnosis may be confused with inflammatory bowel disease resulting in erroneous administration of steroids. Colonoscopy-guided biopsy helps in differentiating amoebiasis from other forms of colitis. Many cases of fulminant amoebic colitis reported from Western countries were misdiagnosed preoperatively as fulminating ulcerative colitis or perforated appendix with peritonitis [17].

Conventional microscopic examination of the stool to look for ova has a low sensitivity (25–60%). Amoebic serology to detect the antigen in the patient's stool and serum is the most sensitive and specific investigation [18]. But in tropical countries, due to the high prevalence of the infection, most of the people have infection at some part of life, so antibodies are usually positive. In such cases, the presence of IgM antibodies points toward recent infection [19]. EIA detects antibodies specific for *E. histolytica* in approximately 95% of patients with extraintestinal amoebiasis, 70% with active intestinal infection, and 10% who are asymptomatic cyst passers, and it has now largely replaced the indirect hemagglutination test and counter immunoelectrophoresis [20] Entamoeba can be cultured on egg-based media but due to the unavailability of techniques, the method is not commonly used for diagnosis.

The majority of the infected patients are asymptomatic, which can be explained by the presence of two morphologically identical species of Entamoeba, *E. dispar*, the more common species, associated solely with the asymptomatic carrier state, and *E. histolytica*, the pathogenic species, which can invade tissues and cause symptomatic disease [21]. The diagnosis of asymptomatic infection on a large scale is difficult. Antigen detection in stools may be useful as an adjunct to microscopic diagnosis in detecting parasites and to distinguish between pathogenic and nonpathogenic infections. Asymptomatic patients should be treated with luminal amoebicides because they can infect others and 4–10% develop the invasive disease within a year if left untreated [22].

CT is the imaging of choice for diagnosis and assessment of complications [23]. Early CT findings of colitis include bowel wall thickening and irregular mucosa that can be seen on CT enteroclysis [10]. All these findings are nonspecific and point only towards an inflammatory pathology of the large bowel. When gangrene sets in, the bowel wall shows pneumatosis intestinalis and no enhancement on CECT. The features of peritonitis may be absent in 20% of cases with colonic perforation, as there might be a slow leak from the bowel and radiographic evidence of pneumoperitoneum may be seen in <50% of cases as the large bowel might perforate into the retroperitoneum [11]. Pathology of the invaded colonic tissue shows transmural inflammation and widespread necrosis, along with large numbers of amoebic trophozoites within the inflammatory exudates [24].

Eggleston et al. [25] and Lubyuski et al. [26] studied multiple cases of large bowel perforation in amoebic colitis and advocated diversion and drainage as the main procedure; mortality rates associated with resection in these two studies were 71% and 83%, respectively. Another study reported a series of 50 adult patients with fulminating amoebic colitis over 20 years and emphasized the importance of aggressive surgical intervention to improve survival. He reported a mortality rate 0f 60% [27]. A study at the University of California reported only one survivor among six patients treated for this disease, many of whom were properly diagnosed and treated [28].

For acute amoebic colitis, once suspected, early diagnosis and aggressive supportive and antiamoebic treatment should be instituted. Management is a bit onerous, as there is a low threshold of suspicion for amoebiasis as a cause of perforation, and the diagnosis is made intraoperatively or after the biopsy report. Intraoperatively, the diseased colon is extensively friable and disintegrates with simple manipulation, as happened in our case [29]. In fulminant colitis, the outcome is poor with mortality ranging from 55% to 87.5%. When perforation and peritonitis complicate acute colitis, this mortality rate increases dramatically to between 75% and 100% in adults [28], peritonitis being the commonest cause of death. Uncomplicated amoebic colitis is readily treated medically and has a mortality rate of less than 0.5% [30]. Early diagnosis and surgical treatment significantly decrease mortality [31]. Resection and anastomosis during the first surgery should always be discouraged as the chances of an anastomotic leak are very high due to inflamed and friable bowel. Also, the ulcers in the distal segment may slough off leading to leaking. A staged surgery in the form of exteriorization of the proximal and distal transected ends as stoma, and bowel reconstruction 3–6 months later, is highly recommended [28]. This will not only remove the diseased segment but also remove the septic foci and prevent further faecal contamination.

## 4. Conclusion

It is important to consider the possibility of FulNAC as an uncommon and grave complication of amoebiasis, especially in tropical countries, where amoebiasis is prevalent. Even with advancements in the field of surgery, the outcome of FulNAC is poor and has not changed much over the decades. Early diagnosis and antiamoebic treatment, along with urgent aggressive surgical resection of the involved segment and exteriorization of the proximal and distal bowel ends, are shown to reduce mortality. During surgery, the entire large bowel must be examined to look for any perforation or impending perforation and to rule out whole bowel involvement.

## Abbreviations

FulNAC: Fulminant necrotising amoebic colitis
BP: Blood pressure
RR: Respiratory rate
OPD: Outpatient department
CT: Computed tomography
CECT: Contrast-enhanced computed tomography
KFT: Kidney function test
ABG: Arterial blood gas
EIA: Enzyme immunoassays.
